# Non-tuberculous mycobacterial disease: progress and advances in the development of novel candidate and repurposed drugs

**DOI:** 10.3389/fcimb.2023.1243457

**Published:** 2023-10-02

**Authors:** Yuzhen Gu, Wenjuan Nie, Hairong Huang, Xia Yu

**Affiliations:** ^1^National Clinical Laboratory on Tuberculosis, Beijing Key Laboratory on Drug-Resistant Tuberculosis, Beijing Chest Hospital, Capital Medical University, Beijing, China; ^2^Tuberculosis Department, Beijing Chest Hospital, Capital Medical University, Beijing, China

**Keywords:** NTM (nontuberculous mycobacteria), antibiotic, inhibitor, NTM pulmonary disease, treatment

## Abstract

Non-tuberculous mycobacteria (NTM) are opportunistic pathogens that can infect all body tissues and organs. In particular, the lungs are the most commonly involved organ, with NTM pulmonary diseases causing serious health issues in patients with underlying lung disease. Moreover, NTM infections have been steadily increasing worldwide in recent years. NTM are also naturally resistant to many antibiotics, specifically anti-tuberculosis (anti-TB) drugs. The lack of drugs targeting NTM infections and the increasing drug resistance of NTM have further made treating these mycobacterial diseases extremely difficult. The currently recommended NTM treatments rely on the extended indications of existing drugs, which underlines the difficulties of new antibiotic discovery against NTM. Another challenge is determining which drug combinations are most effective against NTM infection. To a certain extent, anti-NTM drug development depends on using already available antibiotics and compounds. Here, we aimed to review new antibiotics or compounds with good antibacterial activity against NTM, focusing on their mechanisms of action, *in vitro* and *in vivo* antibacterial activities.

## 1 Introduction

Non-tuberculous mycobacteria (NTM) refer to mycobacteria other than *Mycobacterium leprae* and *Mycobacterium tuberculosis* complex (MTC). NTM prevalence has demonstrated an increasing global trend in the last few decades (Yu et al., 2016; Brode et al., 2017; Lin et al., 2018; Santin et al., 2018; Cowman et al., 2019). In China, the proportion of NTM isolates among the specimen cultures positive for *Mycobacterium* species rose from 4.3% in 1979 to 22.9% in 2010 (Zhou et al., 2020). NTM are present in soil and water and are widely dispersed in the natural world. Most of the more than 200 isolated NTM species are non-pathogenic, whereas approximately 30 are clinically relevant. Although the nosocomial spread of *M. abscessus* (*Mab*) in cystic fibrosis patients was reported, the precise source of the remaining NTM species infection has no conclusive proof of transmission between people, which is assumed to be acquired via environmental exposure (Bryant et al., 2016; Wu et al., 2018).

The resistance of NTM to existing treatments is increasingly becoming an internationally recognized problem (Griffith et al., 2007; Johansen et al., 2020; Griffith and Daley, 2022). These include the most common clinical cases of *Mab* and *Mycobacterium avium* complex (*MAC*), which account for >90% of all documented NTM pulmonary disease (NTM-PD) cases (Yu et al., 2016).The regimens for *Mab* infections contain macrolide antibiotics, including clarithromycin (CLA) and azithromycin. However, most patients respond poorly to this class of antibiotics due to the inducible resistance phenotype that occurs during therapy, which is driven by the macrolide-inducible ribosomal methylase encoded by *erm (41)* (Richard et al., 2020). *Mab* has recently developed increased resistance to CLA, with reported resistance rates of 14%–38% (Broda et al., 2013; Zhuo et al., 2013; Lee et al., 2014). In addition, *Mab* also exhibits resistance to azithromycin (resistance rate of 10%), which is relatively lower than that of CLA (Pasipanodya et al., 2017; Hirama et al., 2020). A meta-analysis shows that the estimated pooled treatment success rate for patients with *MAC* disease was 39% (Xu et al., 2014), similar to the treatment outcomes of extensively drug-resistant tuberculosis (XDR-TB). Macrolides have also been used as the key medication in *MAC* therapy regimens, with a meta analysis indicating a treatment success rate of 60% (95% confidence interval [CI], 55.1%–64.8%) for macrolide-containing regimens in *MAC*-PDs (Kwak et al., 2017). Despite the combination strategy of fluoroquinolones, aminoglycosides, and surgical resection, macrolide-resistant *MAC* lung disease has a poor treatment outcome, resulting in a 1-year all-cause mortality rate of 10% (Park et al., 2019).

**Figure 1:**
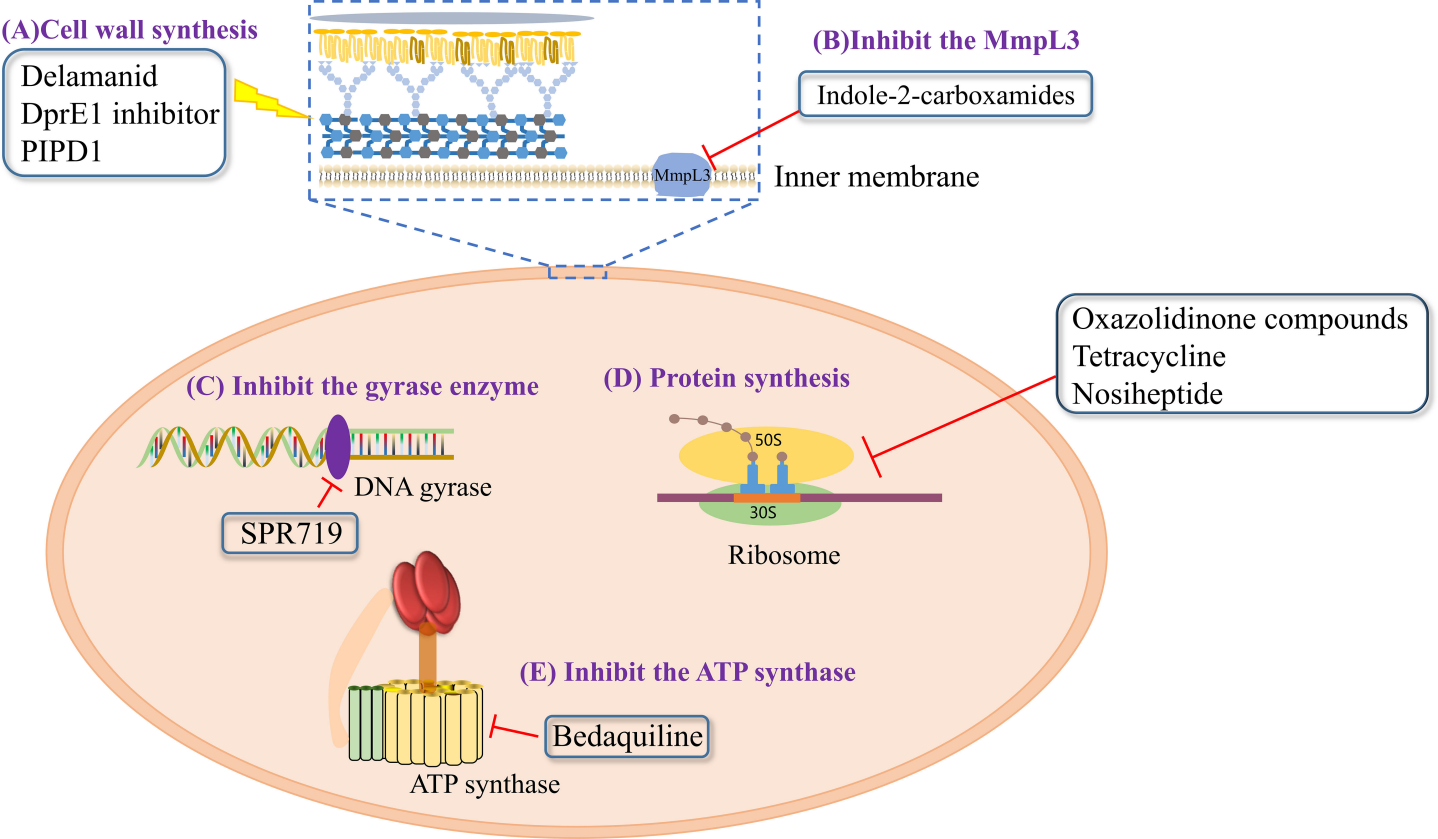
Modes of actions of drugs or compounds against NTM.

However, no drugs have been specifically developed for the increasingly prevalence of NTM infection worldwide. All pharmaceuticals currently recommended by the American Thoracic Society for NTM therapy regimens are derived from the expanded indications of currently available medications. Over the last 50 years of tuberculosis (TB) research, only bedaquiline (BDQ), delamanid (DLM), and pretomanid are the new drugs that have been approved for commercialization by the US Food and Drug Administration (FDA) due to the challenges in developing novel medications. Therefore, maximizing the anti-NTM activity of presently accessible drugs or antibiotics may be useful for developing anti-NTM medications. In this study, we elaborate on new antibiotics or compounds with potent antimycobacterial activity against NTM. Reported drug type, targets, range of concentration, side effects, and structure are summarized in **Table 1** and **Figure 1**.

**Table 1:** Main characteristics of the described new drugs against NTM.

Drug type	Targets	Name	The range of concentration	Side effect	Structure of drugs	References
Oxazolidinone compounds	Attaching to the 23S ribosome and preventing microbial protein synthesis	Linezolid	600 mg QD or BID; Oral; C_max_ for 600 mg orally BID is 21.2 (SD 5.78) mg/L	Headache, nausea and diarrhea, the adverse myelosuppression and hematological disease	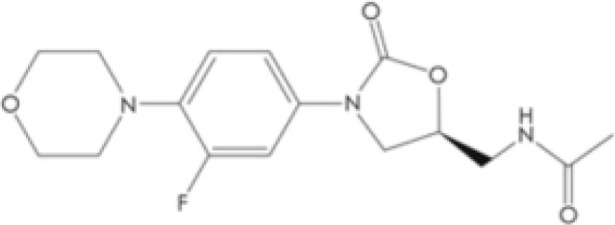	Aguilar Diaz et al., 2023; Burdette and Trotman 2015; Märtson et al., 2021; Yuan et al., 2023
		Tedizolid	200 mg QD; Oral or IV; C_max_ for 200 mg QD is 1.8–2.6 mg/L		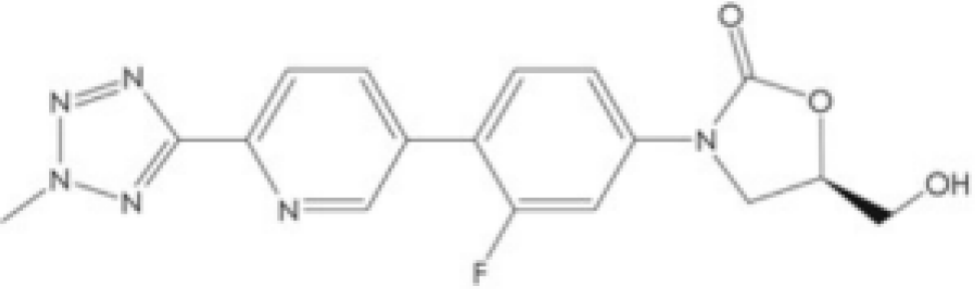	Burdette and Trotman 2015; Cattaneo, Alffenaar, and Neely 2016; Iqbal, Milioudi, and Wicha 2022; Moran et al., 2014; Prokocimer et al., 2013
		Sutezolid	600 mg BID or 1200 mg QD; Oral; C_max_ values increased from 408 ng/mL to 1,550 ng/mL, as dose increased from 300 mg to 1,800 mg		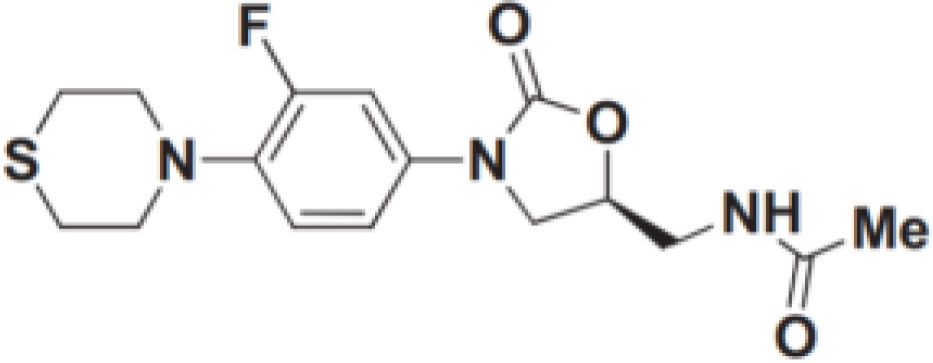	Bahuguna and Rawat 2020; Bruinenberg et al., 2022; Wallis et al., 2014
		Delpazolid	400 mg, 800 mg, 1200 mg QD, or 800 mg BID in a phase IIb trail; Oral or IV; Cmax for IV 800 mg is 12,161.26(SD 2486.04) ng/mL		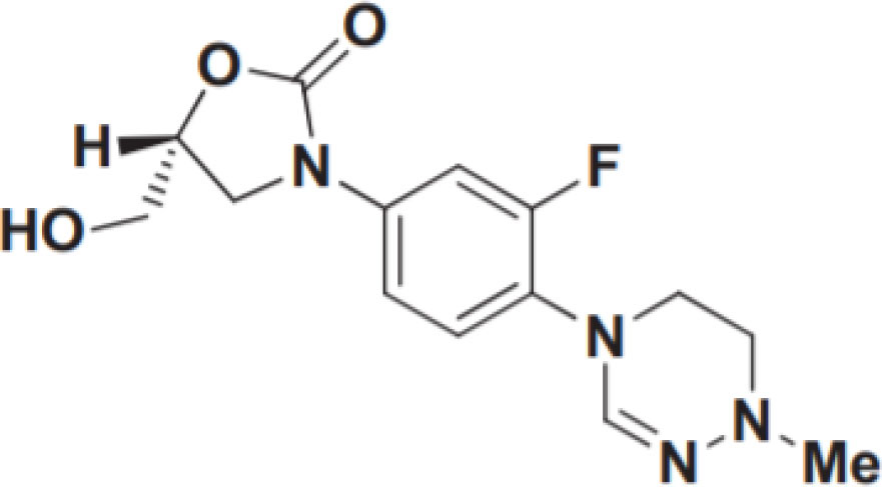	Bahuguna and Rawat 2020; Cho et al., 2019; Dierig et al., 2023
Tetracycline	Blocking protein synthesis by attaching to the 30S ribosomal subunit of the mRNA translation complex	Tigecycline	25–50 mg QD or BID; IV; Cmax for IV 50mg is 0.98 (SD 0.21) μg/ml	Nausea, vomiting and coagulopathy	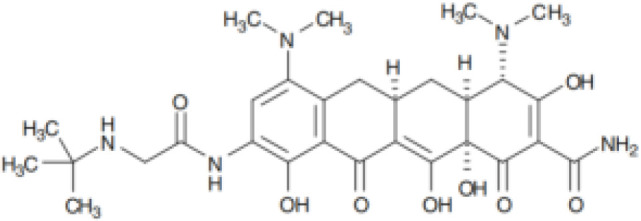	Cui et al., 2019; Daley et al., 2020; Gotfried et al., 2017; Rubinstein and Vaughan 2005
		Eravacycline	1 mg/kg; IV; C_max_ 1,825 ng/ml	Nausea and vomiting	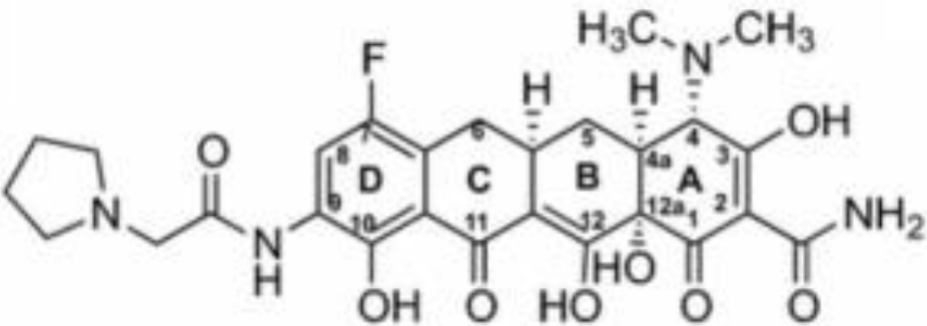	LaPlante et al., 2022; Newman et al., 2019; Zhanel et al., 2016
		Omadacycline	IV 100 mg or Oral 300 mg QD; Cmax 0.5-0.6 mg/L	Nausea and vomiting	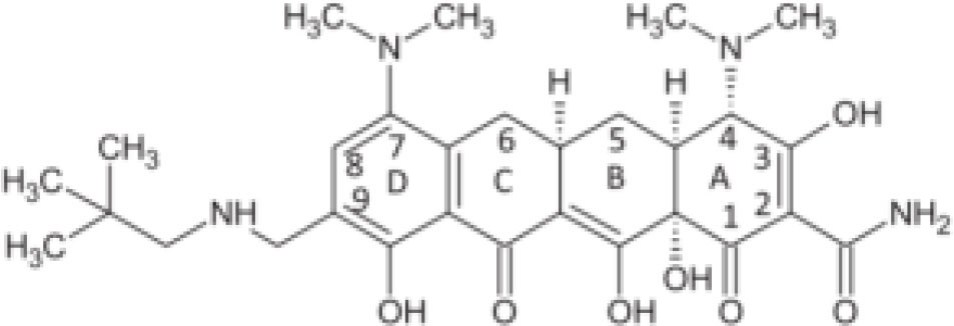	Dougherty et al., 2019; LaPlante et al., 2022; Zhanel et al., 2020
		Sarecycline	60-150mg in acen based on the bodyweight; Oral; C _max,ss_ = 2.09,1.65,1.34mg/L for 100mg across weight groups of 43,70,100kg	Abdominal pain, nausea, diarrhea, sunburn, photosensitivity, headache (nausea)	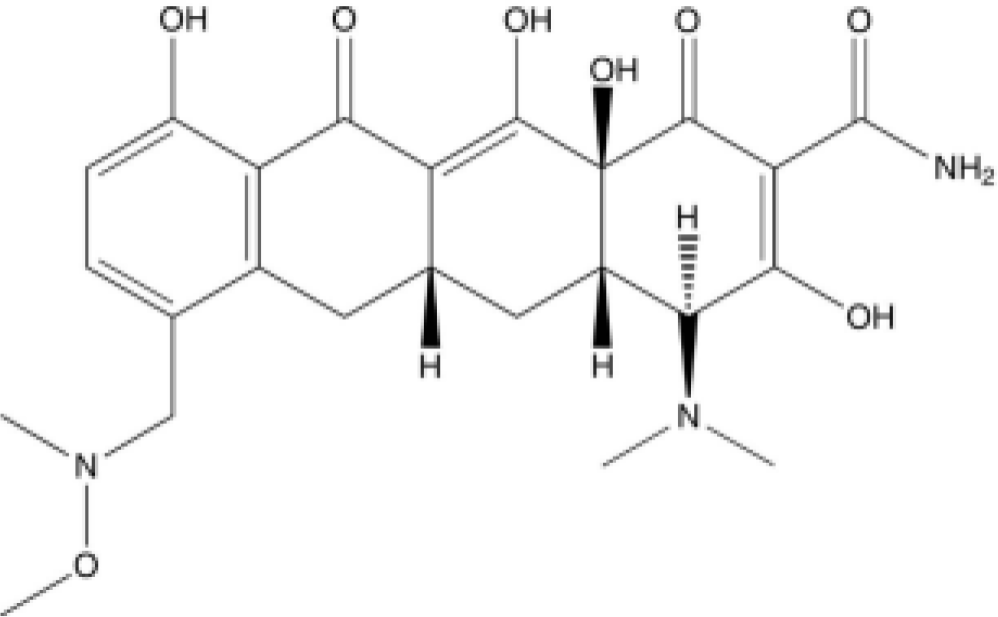	Deeks 2019; Grada et al., 2022; Haidari et al., 2020
Diarylquinoline	Inhibiting ATP synthase (subunit c encoded by atpE) required for oxidative phosphorylation	Bedaquiline	400 mg QD; Oral; Cmax =2.4 (SD 0.8) mg/L	Headache, dizziness, vomiting and arthralgia	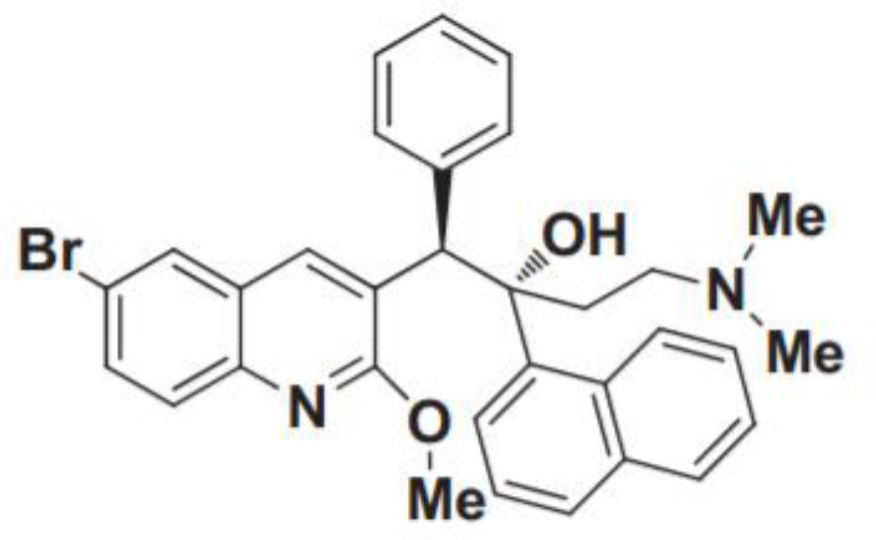	Aguilar Diaz et al., 2023; Bahuguna and Rawat 2020; Khoshnood et al., 2021; Kim et al., 2023; Märtson et al., 2021
		Sudapyridine (WX-081)	In phase III clinical trials		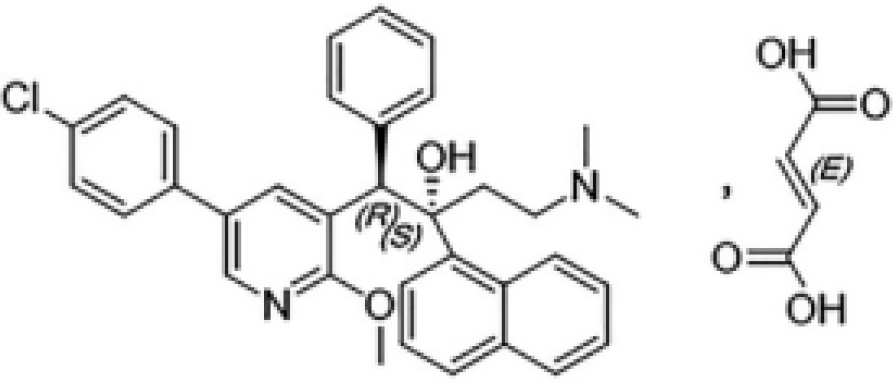	Xiao et al., 2023; Yao et al., 2022
Nitroimidazole	Inhibiting mycolic acid biosynthesis and blocking cell wall production	Delamanid	100 mg BID; Oral; Cmax for 100 mg BID is 414ng/mL, CV39.9%	Nausea, vomiting , and dizziness,potential to cause QT prolongation	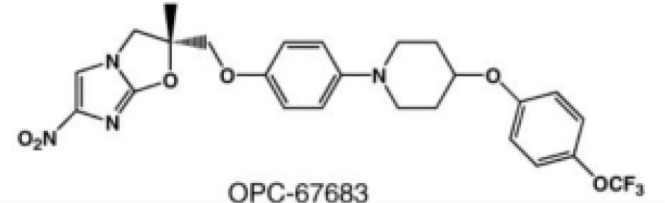	Gler et al., 2012; Matsumoto et al., 2006; Mok et al., 2022; Mudde et al., 2022; Szumowski and Lynch 2015
		Pretomanid	200 mg QD; Oral; Cmax = 1090 (SD 187) ng/mL, CV 17.1%	Vomiting, acne, nausea, headache, musculoskeletal pain, and transaminase elevation	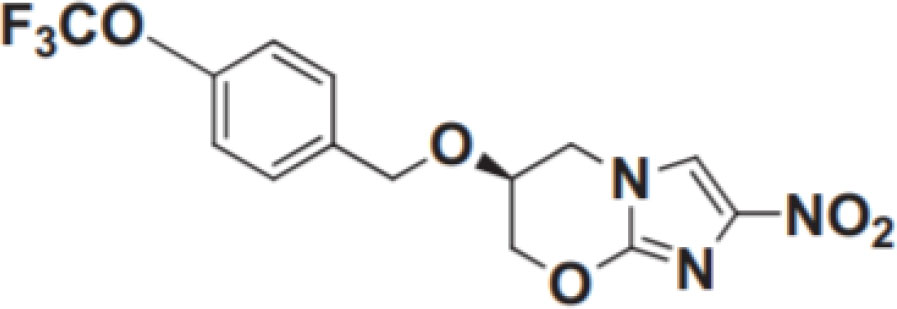	Bahuguna and Rawat 2020; Fekadu et al., 2022; Keam 2019; Liu et al., 2022; Mudde et al., 2022
Clofazimine	Not completely understood		100 mg QD; Oral; Cmax = 0.218 (0.0687–0.4130) mg/L	Discolouration of the skin, gastrointestinal disturbances and QT interval prolongation	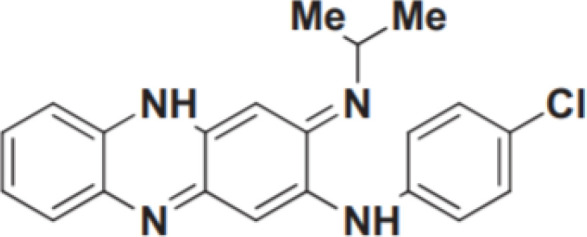	Abdelwahab et al., 2020; Bahuguna and Rawat 2020; Peloquin and Davies 2021; Stadler et al., 2023
DprE1 inhibitor	DprE1 is a crucial enzyme involved in the cell wall synthesis of mycobacterium tuberculosis	OPC-167832	Multiple ascending doses in Phase I/IIa Study; Oral; Cmax for oral 90mg is 391 (SD116) ng/mL	Headache, constipation, and back pain	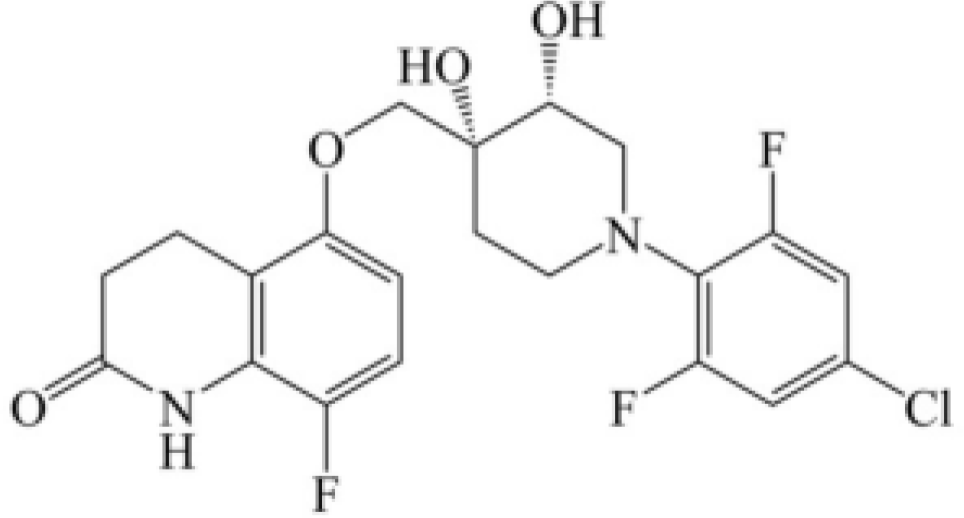	Dawson et al., 2023; Hariguchi et al., 2020; Yadav et al., 2023

C_max_, maximum concentration; C_max.ss_, maximum concentration at steady state; IV, intravenous; SD, standard deviation from the mean; QD, once daily; BID, twice daily.

## 2 Oxazolidinone

*In vitro* and *in vivo* investigations have shown that oxazolidinones are extremely effective at eliminating *M*. *tuberculosis* (Mtb) (Pstragowski et al., 2017; Guo et al., 2021). Their mechanism of action involves attaching to the 23S ribosome, composed of 5S and 23S rRNAs and 36 riboproteins (L1–L36), and blocking the tRNA at the peptidyl transferase center on the ribosomal subunit, thereby preventing microbial protein synthesis (Kadura et al., 2020). The first approved oxazolidinone drug was linezolid (LZD) in 2000. LZD has a wide spectrum of activity against gram-positive bacteria, including methicillin-resistant staphylococci, penicillin-resistant pneumococci, and vancomycin-resistant *Enterococcus faecalis*, as well as drug-resistant TB (Clemett and Markham, 2000). Subsequently, tedizolid (TZD) was approved by the FDA in 2014 as a novel, effective oxazolidinone precursor drug (Zhanel et al., 2015). Another promising drug is sutezolid (SZD, PNU-100480), a thiomorpholine analog of LZD, showing preliminary evidence of superior efficacy against Mtb and readily detectable bactericidal activity in the sputum and blood (Wallis et al., 2014). Similarly, delpazolid (DZD, LCB01-0371) is a novel oxazolidinone analog that exhibits broad-spectrum anti-gram-positive activity *in vitro* and in animal infection models as well as effective protection against *Mab (* Kim et al., 2017).

All four mentioned oxazolidinones demonstrated *in vitro* antimycobacterial activity against 32 rapidly growing mycobacteria (RGM) reference strains, presenting with MICs of ≤8 μg/ml against most species (Wen et al., 2021). Compared with the other oxazolidinones, TZD had the lowest MIC values for *Mab* subsp. *abscessus* and *Mab* subsp. *massiliense*, with MIC_50 _= 1 μg/ml and MIC_90 _= 2 μg/ml for both subspecies (Brown-Elliott and Wallace, 2017; Wen et al., 2021). DZD also exhibited better antimycobacterial activity (4-fold lower MIC values) than LZD against *M. fortuitum* isolates. Additionally, all four oxazolidinones showed *in vitro* antimycobacterial activity against slowly growing mycobacteria (SGM) reference stains, wherein all four drugs had MIC values ≤8 μg/mL against 18 of 20 tested SGM species. Particularly, SZD had the strongest MIC values against *M. intracellulare* (MIC_50 _= 2 μg/mL and MIC_90 _= 4 μg/mL).

Among these four oxazolidinones, LZD and TZD have been used for the therapy of NTM infection, including *MAC*, *M. abscessus, M. fortuitum* and *M. kansasii* (**Table 2**). Consistent with *in vitro* antimycobacterial activity, two of 3 (67%) patients with *M. chelonae* and *M. abscessus* were cured or clinically cured with LZD-containing regimens. Seven of 12 (58%) patients with *M. chelonae* and *Mab* were cured or clinically cured with TZD- containing regimens (Poon et al., 2021). Potential therapeutics of LZD and TZD and the detailed information of case reports using these two drugs are presented in **Table 2** and **Table S1**.

**Table 2:** Potential or existing therapeutics for different species of NTM.

Drug	Potential or existing therapeutics	References
Linezolid	*MAC*	de Melo Carvalho et al. 2020
	*M. abscessus*	Poon et al., 2021; Daley et al., 2020
	*M. fortuitum *	Thorell et al. 2006
Tedizolid	*M. abscessus*	Poon et al., 2021
	*M. chelonae*	Shaw et al., 2021
	*MAC* and *M. kansasii*	Ruth et al., 2020; Yuste et al., 2017
Tigecycline	*M. abscessus*	Daley et al., 2020
	*M. chelonae*	Unai et al., 2013
Omadacycline	*M. abscessus*	Tucker, Droemer, and Condren 2023; Mingora et al., 2023; Rizzo and Moniri 2022
	*M. chelonae*	Rizzo and Moniri 2022
Bedaquiline	*MAC* and *M. abscessus*	Philley et al., 2015; Gil et al., 2021
	*M. fortuitum*	Erber et al., 2020
DLM or Pretomanid	*M. kansasii*	Kim et al., 2019; Zheng et al., 2023
Clofazimine	*M. abscessus*	Shumway et al., 2020; Moguillansky, DeSear, and Dousa 2023; Kaji et al., 2023; Lau et al., 2023; Pinapala et al., 2021; Martiniano et al., 2017; Beech et al., 2023; Cameron et al., 2022; Pfaeffle et al., 2021
	*MAC*	Cariello et al., 2015; Martiniano et al., 2017; Cameron et al., 2022; Pfaeffle et al., 2021
	*M. fortuitum*	Haubrich et al., 2022
	*M. chelonae*, *M. immunogen*, *M. haemophilum*	Pfaeffle et al., 2021

## 3 Tetracycline

Tetracyclines have served as cornerstone antibacterial drugs for over 70 years. This class of drugs blocks protein synthesis by attaching to the 30S ribosomal subunit of the mRNA translation complex to inhibit the binding of aminyl tRNA to the mRNA–ribosomal complex (Yadav et al., 2023). Within this tetracycline group, tigecycline (TGC) is the first and only clinically available glycylcycline (Ng and Ngeow, 2022). Additionally, several new antibiotics from the tetracycline class were recently created to address the drawbacks of TGC, i.e., high rate of adverse gastrointestinal effects and mortality (Heaney et al., 2019). Among them, eravacycline (ERC), a synthetic fluorocycline, was authorized by the FDA in 2018. ERC is injected intravenously to treat difficult intra-abdominal infections caused by antibiotic-resistant bacteria (Anonymous, 2018a). In 2018, the FDA approved another drug called omadacycline (OMC) for managing acute bacterial skin and skin structure infections (ABSSI) and community-acquired bacterial pneumonia (CABP). This antibiotic can be administered orally (PO) or intravenously (IV) once per day (Anonymous, 2018b). Sarecycline (SAC), another oral drug, represents the first narrow-spectrum tetracycline-class antibiotic developed for acne treatment (Anonymous, 2018c). Previous studies have demonstrated strong *in vitro* antimycobacterial activity of TGC against RGM and have recommended using TGC for treating *Mab* infections according to the current guidelines (Wallace et al., 2002; Lerat et al., 2014; Wallace et al., 2014; Haworth et al., 2017; Daley et al., 2020). Except for SAC, the other tetracyclines (i.e., TGC, OMC, and ERC) had MICs of ≤0.5 μg/ml against 27 RGM reference strains. In particular, ERC generally presented the lowest MICs, with MIC_90_ values of 0.25 μg/mL, 0.25 μg/mL, and 0.06 μg/mL against the clinical isolates of *Mab* subsp. *abscessus, Mab* subsp*. massiliense*, and *M. fortuitum*, respectively (Shoen et al., 2019). In the case of TGC and OMC, equivalent *in vitro* inhibitory activities were found against these isolates, in which the TGC showed MIC_90_ values that were lower or equal to those of OMC for *Mab* subsp. *abscessus*, *Mab* subsp*. massiliense*, and *M. fortuitum* (1 μg/mL, 1 μg/mL, and 0.25 μg/mL versus 1 μg/mL, 2 μg/mL, and 2 μg/mL) (Zhang et al., 2023). Furthermore, the *in vitro* antimycobacterial activity of ERC was similar to or better than that of CLA (MIC: 0.0625–2 μg/mL for susceptible strains), with CLA being the core antimycobacterial in the *Mab* treatment regimen (Fujiwara et al., 2021).

Case reported by Frizzell M et al. showed that a patient with *M. chelonae* infection receiving OMC containing regimen was considered clinically improved (Frizzell et al., 2020). Notably, 44 of 95 (46%) patients with *Mab*-PD had 1 or more negative cultures, with 17 of 95 (18%) achieving culture conversion (Mingora et al., 2023). Furthermore, the adverse drug effects (ADEs) were relatively mild, 35 patients (29.9%) experienced direct ADEs, nausea/emesis occurring in 21.4% of patients (**Table S1**). Oral administration, high antimycobacterial activity and relatively low adverse effects make OMC a promising drug for *Mab* and *M. chelonae* infection (**Table 2**).

## 4 Diarylquinoline

Diarylquinoline antibiotics act by inhibiting ATP synthase (subunit c encoded by atpE) required for oxidative phosphorylation (Kim et al., 2023). BDQ was the first diarylquinoline approved by the FDA in 2012 for treating pulmonary multidrug-resistant TB (MDR-TB) (Cox and Laessig, 2014). Recently, an innovative diarylpyridinated drug called sudapyridine (WX-081), which is formed by substituting the bromoquinoline of BDQ with a 5-phenylpyridine, was applied at the clinical development stage in 2018 and revealed better safety on QTc intervals. This drug has been included in phase III clinical trials as a TB treatment (JYP0081M301) in China since 2022 (Huang et al., 2022).

Based on the similarities of atpE within the genus *Mycobacterium*, several studies have evaluated the inhibitory activities of BDQ against different NTM species. Our previous study showed that BDQ possessed consistently strong antimycobacterial activity against almost all 18 SGM species tested (all MICs were far below 1 μg/ml), while BDQ also exhibited strong *in vitro* antimycobacterial activity against the tested RGM reference strains, with most MIC values ≤2 μg/mL (Aguilar-Ayala et al., 2017; Martin et al., 2019; Yu et al., 2019). Considering the bimodal distributions of BDQ MICs, the tentative epidemiological cutoff (ECOFF) values for SGM and RGM were set as 1 μg/mL and 2 μg/mL, respectively (Pang et al., 2017). In a Moscow study, the MIC_50_ and MIC_90_ values of BDQ were 0.015 μg/ml and 0.12 μg/ml for *M. avium*, and 0.007 μg/ml and 0.06 μg/ml for *M. intracellulare*, respectively. Consequently, the preliminary ECOFF values were defined as 0.12 μg/ml and 0.06 μg/ml for *M. avium* and *M. intracellulare*, respectively (Pidot et al., 2021). In South Korea, although the MIC_50_ and MIC_90_ values of BDQ were extremely low against Mab subsp. *abscessus* and Mab subsp. *massiliense* isolates (MIC_50 _= 0.062 μg/ml and MIC_90 _= 0.125 μg/ml), these values were higher than those for *MAC* (≤0.016 μg/ml) and *M. kansasii* isolates (≤0.016 μg/ml) (Kim et al., 2019). For sudapyridine, the BDQ analog, the MIC values against most clinical isolates of NTM were at least one dilution higher than the MICs of BDQ for six frequently isolated NTM species (Zhu et al., 2022).

There are several studies utilizing BDQ containing regimens for NTM infection (Philley et al., 2015; Erber et al., 2020; Gil et al., 2021), including *Mab*, *MAC* and M. *fortuitum* (**Table S1**). A small preliminary report indicated the potential clinical and microbiological activity of BDQ in patients with advanced *MAC* (n = 6) or *Mab* (n = 4) lung disease, with 60% of patients (six of 10) demonstrating a microbiologic response (Philley et al., 2015). Due to strong antimycobacterial activity of BDQ, it seems to be used for all pathogenic NTM, especially for refractory *Mab* and *MAC* infection (**Table 2**).

## 5 Nitroimidazole

Nitroimidazole is a class of novel antimycobacterial agents that eradicate active Mtb by inhibiting mycolic acid biosynthesis and blocking cell wall production (Lewis and Sloan, 2015; Zhang et al., 2019). DLM, a bicyclic nitroimidazole, was initially approved by the European Medicines Agency in 2014 for pulmonary MDR-TB in adult. Pretomanid was the second bicyclic nitroimidazole drug that received its approval in the USA in 2019 for treating adults with pulmonary XDR-TB or treatment-tolerant or non-responsive MDR-TB (Gils et al., 2022).

Our earlier research demonstrated that DLM had highly variable antimycobacterial activity against 19 tested SGM species, with MICs of <0.25 μg/ml in 11 species. In contrast, DLM displayed no significant inhibitory activity against most tested RGM species, with 28 of the 33 tested strains having MICs >32 μg/ml (Yu et al., 2019). Except for a few strains with MICs ≤1 μg/mL, DLM did not possess a strong inhibitory effect against *M. intracellulare* and *M. abscessus*. Similarly, Philley JV et al. found that DLM had extremely high MIC_90_ (>16 μg/ml) values against *MAC* and *Mab* complex. Compared to the MICs of DLM for those NTM, relatively low MIC_50_ (0.25 μg/mL) and MIC_90_ (1 μg/ml) values were observed against *M. kansasii* (Kim et al., 2019). Similar to DLM, pretomanid expressed high *in vitro* antimycobacterial activity against *M. kansasii* (MIC = 1.71 μg/mL) and moderate antimycobacterial activity against *M. xenopi*, with an MIC of 3.84 μg/mL (Zheng et al., 2023). Although high inhibitory potency was detected in DLM and pretomanid against *M. kansasii in vitro*, DLM or pretomanid containing regimens has not been used for the therapy of NTM infection in patients to date. Thus, DLM or pretomanid may be a potential efficacy drug for drug resistant *M. kansasii* (**Table 2**).

## 6 CFZ

CFZ, a traditional hydrophobic riminophenazine, has been prescribed for leprosy management since the 1950s. Although the exact mechanism of CFZ-mediated antimycobacterial activity remains undeciphered, the cell membrane may be the primary target (Cholo et al., 2017; Mirnejad et al., 2018; Stadler et al., 2023). The antimycobacterial activity of this riminophenazine covers a wide range, extending from anti-leprosy to NTM efficacy. Our prior investigation showed that CFZ had good activity against reference strains and clinical isolates of varied SGM species, with MICs well below 1 μg/ml for most strains (Luo et al., 2018). Furthermore, most clinical isolates of *Mab* and *M. fortuitum* had MICs >2 μg/ml. However, the ECOFF values for *M. kansasii*, *M. avium* and *M. intracellulare* were defined as 0.5 μg/ml, 1 μg/mL, and 2 μg/ml, respectively, based on their MIC distributions (Luo et al., 2018). In a study of the *Mab* clinical isolates obtained from patients with cystic fibrosis, 70% of the isolates presented with an MIC of ≤1.5μg/mL (Banaschewski et al., 2019).

CFZ containing regimens has been used for a wide range of NTM infection with about 50% favourable treatment outcomes, including *Mab*, *MAC*, *M. chelonae and M. haemophilu (* Martiniano et al., 2017; Pfaeffle et al., 2021) (**Table S1**). A prospective cohort study was performed in 36 NTM infection patients treated with CFZ, 22 (58%) out of 36 patients had treatment success, including 12 of 19 (63%) with *Mab (* Martiniano et al., 2017). Furthermore, a phase II clinical trial of CFZ evaluating its efficacy in *MAC*-PD treatment is underway (https://clinicaltrials.gov/study/NCT02968212) (Ito et al., 2022).Thus, current data supported CFZ in the treatment of NTM like *Mab*, *MAC* and *M. chelonae* (**Table 2**).

## 7 Compound drugs

### 7.1 DprE1 inhibitor

In 2009, the decaprenylphosphoryl-beta-D-ribose oxidase (DprE1) enzyme was identified as a novel anti-TB drug target owing to its crucial role in *mycobacteria* and its location in the bacterial cell wall (Morrisette et al., 2021; Edwards and Field, 2022). Currently, several DprE1 inhibitors are enrolled in clinical trials, including BTZ-043, macozinone (MCZ, PBTZ169), OPC-167832 and TBA-7371 (Edwards and Field, 2022).There is no *in vitro* activity of BTZ-043 and TBA-7371 against NTM. PBTZ169 had poor activity against *MAC* and *Mab* isolates with MIC_90_ of >32 μg/mL (Shi et al., 2018). Surprisingly, after 4 weeks treatment in mice, PBTZ169 showed an average 3.33 and 2.29 log_10_ CFU reductions in the lung against *Mab* and *M. chelonae* infection (Zheng et al., 2023). Among these DprE1 inhibitors, OPC-167832 displayed superior efficacy even at low doses in a mouse TB model using as monotherapy or combined treatment with other anti-TB drugs (Robertson et al., 2021; Tasneen et al., 2022). Additionally, other studies have revealed that OPC-167832 harbored substantial activities against *Mab in vitro*, with MICs ranging from 5.2 μM (2.37 μg/mL) to 15 μM (6.85 μg/mL) (Hariguchi et al., 2020; Sarathy et al., 2022), which made it a promising candidate for *Mab* infection.

### 7.2 SPR720

SPR720, is a prodrug that is converted to SPR719, is a novel aminobenzimidazole that inhibits the gyrase enzyme by targeting its ATPase subunits (Brown-Elliott et al., 2018; Pennings et al., 2021). Brown-Elliott BA et al. showed that SPR719 had MIC_50_ values of 0.06–4 μg/mL for 93 RGM isolates, whereas the 41 *MAC* strains were associated with MIC_90_ and MIC_50_ values of ≤2μg/mL and ≤1μg/mL, respectively (Brown-Elliott et al., 2018). Another study of SPR719 demonstrated its activity against clinically relevant *mycobacteria* in mouse models of *M*. *avium* and *Mab* infections (Talley et al., 2021). Furthermore, a phase I trial showed that a once-daily oral administration of SPR720 (a phosphate prodrug of SPR719) could provide predicted therapeutic exposures of SPR719. Recently, SPR720 was granted the Investigational New Drug status by the FDA as a novel oral agent for pulmonary NTM infections and was recently enlisted in a phase IIa clinical trial for these infections.

### 7.3 GSK286

GSK286 is a Leucyl*–*tRNA synthetases (LeuRS) inhibitor with potent *in vitro* activity against Mtb and a Phase IIa clinical trials for systemic use against tuberculosis is underway (Bouz and Zitko, 2021). GSK286 showed potent antibacterial activity against *Mab*, with MICs of ≤0.25 μg/mL, yielding a MIC_90_ of 0.063μg/mL. In contrast, it was not effective against *MAC* with the MIC_50_ and MIC_90_ values were >8μg/mL (Dong et al., 2020).

### 7.4 Indole-2-carboxamide derivatives

Mycolic acid transporter protein MmpL3 is inhibited by a wide range of structurally unrelated small molecules (Raynaud et al., 2020). Among these MmpL3 inhibitors, indole-2-carboxamides (ICs) block the export of alginate monomycolate to the outer membrane and can significantly inhibit bacterial growth. ICs have been identified as a novel chemical scaffold exhibiting good preclinical efficacy against Mtb and NTM pathogens. Several novel ICs with MIC values of 0.0039–8 µg/mL against NTM have been recently reported (Pandya et al., 2019). Of these, two lead IC compounds (compounds 5 and 25) showed effective bactericidal activity against *Mab in vitro* (MIC = 0.125 µg/mL) (Pandya et al., 2019). Considering these findings, the chemical inhibition of MmpL3 can be hypothesized to enhance the efficacy of other drugs owing to their crucial role in modulating cell wall structure and composition.

### 7.5 Piperidinol-based compounds

Piperidinol-based compounds (PIPD1) strongly inhibit the transport of trehalose monomycolate, thereby disrupting the mycolylation of arabinogalactan (Dupont et al., 2016; Pandya et al., 2019). PIPD1 has been reported to possess potent activity against several mycobacterium species, including *Mab*, *M. chelonae* and *M. smegmatis* (MIC <1 μg/mL). In particular, PIPD1 exhibited MICs of 0.125 μg/mL against all 32 *Mab* strains, while its MBC_99_ values of 0.125–0.5 µg/mL indicated bactericidal activity (Degiacomi et al., 2019). Additionally, PIPD1 was found to have high levels of antimycobacterial activity in THP-1 macrophages, with decreasing 2 log_10_ CFU at a concentration of 6 μg/mL (48 × MIC). Moreover, PIPD1 administration (3 μg/mL of PIPD1 for 72 h) in a *Mab*-infected zebrafish model reduced bacterial load and increased the survival of the infected embryos (Dupont et al., 2016).

In summary, identifying novel anti-NTM drugs is of vital importance in the face of increasing global NTM infections. Anti-TB drugs, such as BDQ and CFZ have shown good *in vitro* anti-NTM activity and have been proposed for clinical use. Compounds, including OPC-167832, have also shown good *in vitro* antibacterial activity in clinical trials involving common pathogenic NTM. Therefore, these drugs or compounds have potential for in NTM treatment to improve patient outcomes. Although *in vitro* and preclinical trials have detected many promising compounds with potential therapeutic effects against NTM infection, clinical trials are urgently required to investigate their efficacy in NTM disease management. In addition, phage therapy for NTM infection also acquired favorable outcomes with exceptional safety profiles and no evidence of phage resistance was observed, which makes it a promising potential therapy. Thus, we believe that the increasing attention on NTM diseases should result in increased efforts on relevant drug discovery necessary to close the gaps in NTM treatment.

## Author contributions

YG and XY drafted the manuscript. XY, HH and WN designed the study and revised the manuscript critically for important intellectual content. All authors contributed to the article and approved the submitted version.
