# Supplementary material for: Non-tuberculous mycobacterial disease: progress and advances in the development of novel candidate and repurposed drugs
Source: Front Cell Infect Microbiol. 2023 Oct 2;13:1243457. doi: 10.3389/fcimb.2023.1243457 (PMC10577331; doi:10.3389/fcimb.2023.1243457)
Supplement: Supplementary file 1 [file Table_1.docx]

Table S1 Case reports of NTM infection using new antimycobacterial drug

| Drug | Number of patients | Species identification | Companion Drugs | Treatment outcomes | Adverse effect | References |
| --- | --- | --- | --- | --- | --- | --- |
| Linezolid | 1 | *M. chimaera* | amikacin, clarithromycin, ethambutol,  moxifloxacin,  rifabutin | Slow but gradual improvement | Linezolid-related severe myelosuppression | de Melo Carvalho et al., 2020 |
|  | 3 | *Mab* and  *M. chelonae* | amikacin, azithromycin, clarithromycin, imipenem, tigecycline | 2 of 3 (67%) patients were cured or clinically cured | Cytopenia and thrombocypetnia | Poon et al., 2021 |
| Tedizolid | 1 | *M. chelonae* | clarithromycin, tobramycin, imipenem/cilastatin | Skin lesions regressed and remained in remission | Occasional, milder recurrence of similar skin lesions and peripheral neuropathy | Shaw et al., 2021 |
|  | 1 | *MAC* and  *M. kansasii* | linezolid, ethambutol, azithromycin | Both clinical and radiological (chest CT scan) improvement | Decline of haemoglobin | Yuste et al., 2017 |
|  | 12 | *Mab* and  *M. chelonae* | azithromycin, bedaquiline, clofazimine, imipenem, tigecycline | Seven of 12 (58%) patients were cured or clinically cured | 1 patient experienced gastrointestinal side effects, and three patients experienced nausea/vomiting, cytopenia | Poon et al., 2021 |
| Tigecycline | 1 | *M. chelonae* | tobramycin, imipenem, moxifloxacin, clarithromycin, vancomycin | Discharged without relapse for one year | The patient tolerated the treatment of tigecycline for two weeks | Unai et al., 2013 |
|  | 52 | *Mab* and  *M. chelonae* | clarithromycin, cefoxitin, imipenem, linezolid, amikacin | 48.1% (25/52) patients were considered clinically improved | Twelve patients had serious ADEs included vomiting/nausea, epigastric pain, nausea and anorexia, ileus and acute pancreatitis, hypoglycaemia and acute pancreatitis, liver dysfunction, junctional arrhythmia, facial oedema and venous thrombosis | Wallace et al., 2014 |
| Omadacycline | 1 | *M. chelonae* | steroids, prednisone | Clinical improvement | The patient tolerated the treatment of omadacycline for 4 months | Frizzell, Carr, and Brust 2020 |
|  | 12 | *Mab* | amikacin, imipenem, linezolid/tedizolid, azithromycin, clofazimine | Clinical success was achieved in nine cases | 3 patients experienced ADEs including GI ADE (nausea/vomiting/diarrhea) and abnormal hepatic function | Morrisette et al., 2021 |
|  | 117 | Mab | amikacin, azithromycin, carbapenem, cefoxitin, clarithromycin, clofazimine | 44 of 95 (46%) patients had 1 or more negative cultures, with 17 of 95 (18%) achieving culture conversion | 35 patients (29.9%) experienced direct ADEs, nausea/emesis occurring in 21.4% of patients. Other ADEs included abnormal hepatic function, rash, anemia, eosinophilia, leukopenia, esophagitis, diarrhea, and weight loss | Mingora et al., 2023 |
| Bedaquiline | 1 | *M. fortuitum* | cilastin, imipenem, levofloxacin, mikacin | Cured | Apart from persisting nausea, the patient did not report any notable treatment-related side-effects | Erber et al., 2020 |
|  | 2 | one *Mab*, one *MAC* | amikacin, clofazimine, ethambutol, meropenem, tedizolid | Cured or clinical cured | Bedaquiline was well tolerated | Gil et al., 2021 |
|  | 10 | six *MAC* and four *Mab* | amikacin, azithromycin, ethambutol, rifabutin, streptomycin, tigecycline | 60% of patients (6/10) had a microbiologic response, with 50% (5/10) having one or more negative cultures | Common side effects included nausea (60%), arthralgias (40%), and anorexia and subjective fever (30%) | Philley et al., 2015 |
| Clofazimine | 5 | *MAC* | amikacin, azithromycin, ethambutol, rifabutin, ciprofloxacin clarithromycin clofazimine | 2 of 5 patients achieved microbiological clearance | 80% (4/5) patients experienced ADEs. 3 patients experienced a diffuse brownish skin discoloration, one patient had severe GI disturbances | Cariello et al., 2015 |
|  | 39 | *Mab* (21/52),  *MAC* (18/52),  *M. chelonae*(3/52), *M. immunogen* (2/52),  *M. haemophilum* (1/52), mixed NTM infection (7/52)^b^ | amikacin, azithromycin, ethambutol, tigecycline, rifampin, imipenem | Treatment success was achieved in 58 % (21/36) patients ^a^, including 12 of 19 (63 %) with *Mab* | 15 (39 %) patients reporting potential side effects, gastrointestinal upset in 12 patients (31 %), followed by skin pigmentation in 6 (15 %) | Pfaeffle et al., 2021 |
|  | 112 | *Mab*(54/112), *MAC*(41/112), mixed and unidentified NTM(17/112) | amikacin, ethambutol, imipenem, moxifloxacin, macrolide, rifamycin | 50% patients (41/82) with pulmonary disease converted to negative cultures. 42% (11/26) of patients with *MAC*, 50% (18/36) with *Mab*, and 33% (4/12) with > 1 NTM species were responders. In patients with extrapulmonary wound disease, 9 of 10 (90%) experienced wound healing | Thirteen patients (12%) reported no clinical ADEs. Ninety-eight (88%) reported at least one ADE. Seventy-four patients (66%) reported skin-related ADEs, and Sixty-two (55%) reported GI ADEs | Martiniano et al., 2017 |

ADE, Adverse Drug Event; GI, gastrointestinal

^a^In total 39 patients included 1 was lost to follow-up and 2 people received CFZ for less than the minimal exposure time for inclusion. Analyze 36 patients with evaluable outcomes.

^b^Patients could have received multiple cultures; thus, total cultures will be greater than N = 39. Total number of species will be greater than N = 39 because of Mixed NTM infection - indicates that multiple mycobacterial species were identified in a single patient.
